# Boundary Element and Sensitivity Analysis of Anisotropic Thermoelastic Metal and Alloy Discs with Holes

**DOI:** 10.3390/ma15051828

**Published:** 2022-02-28

**Authors:** Mohamed Abdelsabour Fahmy, Mohammed Owaidh Alsulami

**Affiliations:** 1 Department of Mathematics, Jamoum University College, Umm Al-Qura University, Makkah 25371, Saudi Arabia; 2 Faculty of Computers and Informatics, Suez Canal University, New Campus, Ismailia 41522, Egypt; 3 Department of Mathematical Sciences, Faculty of Applied Sciences, Umm Al-Qura University, Makkah 24381, Saudi Arabia; s44286044@st.uqu.edu.sa

**Keywords:** boundary element method, sensitivity, metal, alloy, anisotropic, thermoelasticity

## Abstract

The main aim of this paper was to develop an advanced processing method for analyzing of anisotropic thermoelastic metal and alloy discs with holes. In the boundary element method (BEM), the heat impact is expressed as an additional volume integral in the corresponding boundary integral equation. Any attempt to integrate it directly will necessitate domain discretization, which will eliminate the BEM’s most distinguishing feature of boundary discretization. This additional volume integral can be transformed into the boundary by using branch-cut redefinitions to avoid the use of additional line integrals. The numerical results obtained are presented graphically to show the effects of the transient and steady-state heat conduction on the quasi-static thermal stresses of isotropic, orthotropic, and anisotropic metal and alloy discs with holes. The validity of the proposed technique is examined for one-dimensional sensitivity, and excellent agreement with finite element method and experimental results is obtained.

## 1. Introduction

Thermoelastic analysis is a critical topic in engineering that has sparked a lot of attention in recent years. Thermoelastic research can be carried out using experimental, analytical, and numerical solutions. Only problems with simple geometry and specified boundaries can be solved analytically. To solve problems with complicated boundaries, numerical methods such as the finite element method (FEM) or the boundary element method (BEM) must be utilized. 

When thermal effects are considered, several methods for solving the volume integral equation in the boundary element formulation have been presented over the years [1,2,3]. These methods include the dual reciprocity method [4] and multiple reciprocity method [5], particular integral boundary element method [6], and the exact boundary integral transformation method (EBITM) [7]. The EBITM is the most attractive of these boundary element methods since it maintains the BEM’s notion of border discretization without any additional internal treatments or numerical approximations. The EBITM has been successfully employed to transform the volume integral to the boundary in isotropic thermoelasticity [8] and anisotropic thermoelasticity [9].

The main purpose of the considered boundary element analysis is to convert the additional volume integral into the boundary by using branch-cut redefinitions to avoid utilizing additional line integrals.

## 2. Formulation of the Problem

The thermoelastic field is estimated by solving the elastostatic boundary integral equation with associated thermal data on a region Ω bounded by a surface S, generated first by the boundary element analysis of the following heat conduction equations:

(1) $$k_{ij}\left(x_{1},x_{2}\right)\Theta_{,ij}=c\rho \dot{\Theta },\;k_{ij}=k_{ji}\;,\;{\left(k_{12}\right)}^{2}-k_{11}k_{22}<0,\;\left(i,j=1,2\right),$$

where $k_{ij}$, c, ρ, and Θ are anisotropic thermal conductivity coefficients, specific heat capacity, density, and temperature, respectively.

The plane-stress constitutive equations, which describe the relationship between stress $\sigma_{ij}$ and strain $\varepsilon_{ij}$ for a homogeneous, anisotropic solid in the $x_{1}-x_{2}$ plane ($\sigma_{13}=\sigma_{23}=\sigma_{33}=0$), can be expressed as follows:

(2) $$\begin{array}{c} \left\{\begin{array}{c} \sigma_{11}\\ \sigma_{22}\\ \sigma_{12} \end{array}\right\}=\left[\begin{array}{ccc} c_{11} & c_{12} & c_{16}\\ c_{12} & c_{22} & c_{26}\\ c_{16} & c_{26} & c_{66} \end{array}\right]\left\{\begin{array}{c} \varepsilon_{11}\\ \varepsilon_{22}\\ 2\varepsilon_{12} \end{array}\right\},\\ \left\{\begin{array}{c} \varepsilon_{11}\\ \varepsilon_{22}\\ 2\varepsilon_{12} \end{array}\right\}=\left[\begin{array}{ccc} a_{11} & a_{12} & a_{16}\\ a_{12} & a_{22} & a_{26}\\ a_{16} & a_{26} & a_{66} \end{array}\right]\left\{\begin{array}{c} \sigma_{11}\\ \sigma_{22}\\ \sigma_{12} \end{array}\right\}, \end{array}$$

where $c_{mn}$ and $a_{mn}$ are the stiffness and compliance constants, respectively.

The generalized variable that describes the field point $Q\left(x_{1},x_{2}\right)$ position can be expressed as

(3) $$z_{j}=\left(x_{1}-x_{p1}\right)+\mu_{j}\left(x_{2}-x_{p2}\right),$$

where $\mu_{j}$ is the material complex constant, and $\left(x_{p1},\;x_{p2}\right)$ are the global coordinates of the source point P.

## 3. Boundary Element Implementation

In the presence of the thermal effect of Equation (1) in Equation (2), we can write the following boundary integral equation [10]:

(4) $$\Theta \left(P\right)+\overset{}{\underset{S}{\int }}\left(\Theta^{*}q-q^{*}\Theta \right)dS=\overset{N}{\underset{q=1}{\sum }}\left(\Theta^{q}\left(P\right)+\overset{}{\underset{S}{\int }}\left(\Theta^{*}q^{q}-q^{*}\;\Theta^{q}\right)dS\right){\bar{\alpha }}^{q},$$

where ${\bar{\alpha }}^{q}$ are unknown coefficients, n is the outward unit normal vector, q is the heat flux vector, $\Theta^{q}\;\mathrm{and}\;q^{q}$ are particular solutions, and $\Theta^{*}\;\mathrm{and}\;q^{*}$ are anisotropic fundamental solutions.

Equation (4) can be expressed as the following linear algebraic system [11]:

(5) $$\left[H\right]\left\{\Theta \right\}=\left[G\right]\left\{\frac{\partial \Theta }{\partial n}\right\},$$

where H and G are nonsymmetric and symmetric matrices, respectively.

The thermoelastic field is estimated in a progressively coupled technique by solving the elastostatic BIE with accompanying thermal data, generated first by the boundary element solution of heat conduction.

The boundary integral equation based on the thermal effect can now be written as

(6) $$c_{ij}\left(P\right)u_{i}\left(P\right)=\overset{}{\underset{S}{\int }}U_{ij}^{*}\left(P\cdot Q\right)t_{i}\left(Q\right)dS-\overset{}{\underset{S}{\int }}T_{ij}^{*}\left(P.Q\right)u_{i}\left(Q\right)dS\;+\overset{}{\underset{S}{\int }}\gamma_{ik}n_{k}\Theta \left(Q\right)U_{ij}^{*}\left(P,Q\right)dS\;-\overset{}{\underset{\Omega }{\int }}\gamma_{ik}\Theta_{,k}\left(Q\right)U_{ij}^{*}\left(P,q\right)d\Omega ,$$

where $c_{ij}$, $u_{i}$, $t_{i}$, $\gamma_{ik}$, $U_{ij}^{*},$ and $T_{ij}^{*}$ are the free term, displacements, tractions, thermal moduli, displacement fundamental solutions, and traction fundamental solutions, respectively.

The volume integral is represented by

(7) $$V_{j}=-\overset{}{\underset{\Omega }{\int }}\gamma_{ik}\Theta_{,k}\left(Q\right)U_{ij}^{*}\left(P,q\right)d\Omega$$

According to Lekhnitskii [12], the fundamental solutions are

(8) Uij*P,Qq=2ReβikAjklnzk,

(9) T1j*P,Q=2n1Reμk2Ajkzk−2n2ReμkAjkzk

(10) $$\begin{array}{c} T_{2j}^{*}\left(P,Q\right)=-2n_{1}Re\left\{\frac{\mu_{k}A_{jk}}{z_{k}}\right\}+2n_{2}Re\left\{\frac{A_{jk}}{z_{k}}\right\} \end{array}.$$

where $\beta_{ik}$ and $A_{jk}$ are complex constants, and Re⋅ is the real value of the variable between the brackets.

The volume integral in Equation (7) should be redefined in the transformed domain $\hat{\Omega }$ as follows:

(11) $$V_{j}=-\overset{}{\underset{\Omega }{\int }}\gamma_{-ik}\Theta_{-,k}\left(Q\right)U_{-ij}^{*}\left(P,q\right)d\hat{\Omega }$$

in which

(12) $$\mathrm{style}\;\gamma_{-ik}=\left(\begin{array}{cc} \gamma_{11} & \frac{-\gamma_{11}K_{12}+\gamma_{12}K_{11}}{\sqrt{\Delta }}\\ \gamma_{21} & \frac{-\gamma_{21}K_{12}+\gamma_{22}K_{11}}{\sqrt{\Delta }} \end{array}\right),$$

where $\Delta =k_{11}k_{22}-k_{12}>0$

Then, the volume integral in Equation (11) can be written as follows [13]:

(13) $$V_{j}=\overset{}{\underset{s}{\int }}\left[\left(\gamma_{-ik}Q_{-ijk,t}^{*}\Theta -\gamma_{-ik}Q_{-ijk}^{*}\Theta_{-,t}\right)n_{-t}-\gamma_{-ik}U_{-ij}^{*}\Theta n_{-k}\right]d\hat{S}$$

where

(14) Q−ijk*=2ReβimAjmμ−kmμ−1m2+μ−2m2z−mlnz−m,

(15) Q−ijk,t*=2ReβimAjmμ−kmμ−tmμ−12+μ−221+lnz−m,

and

(16) $$\begin{array}{c} z_{-m}=\mu_{-jm}\left({\hat{x}}_{j}-{\hat{x}}_{pj}\right) \end{array},$$

(17) $$\begin{array}{c} \mu_{-mn}=\left(\begin{array}{cc} \frac{K_{11}+\mu_{1}K_{12}}{\sqrt{\Delta }} & \frac{K_{11}+\mu_{2}K_{12}}{\sqrt{\Delta }}\\ \mu_{1} & \mu_{2} \end{array}\right) \end{array}.$$

The above transformation can be valid upon adding the line integral as follows:

(18) $$\begin{array}{l} V_{j}=\int_{s}[(\gamma_{-ik}Q_{-ijk,t}^{*}\Theta -\gamma_{-ik}Q_{-ijk}^{*}\Theta_{-,t})n_{-t}-\gamma_{-ik}U_{-ij}^{*}\Theta n_{-k}]d\hat{S}\\ \;\;+\sum_{n=1}^{m}\int_{L_{2n-1}}^{L_{2n-2}}L_{j}\left(\zeta_{1}\right)d\zeta_{1}, \end{array}$$

where $\zeta_{1}=\left(x_{1}-x_{p1}\right)$ is the local coordinate of P, and $\left(L_{1},L_{2}\right),\;\left(L_{3},L_{4}\right),ߪ,\;\left(L_{2n-1},L_{2n-2}\right)$ are integral intervals of all source points where branch-cut lines intersect the domain.

Furthermore, $L_{j}$ is expressed as

(19) Lj=4πΘγ−ikK12K11ImβipAjpμ−1pμ−kpμ−1p2+μ−2p2+ΔK11ImβipAjpμ−2pμ−kpμ−1p2+μ−2p2 −4πγ−ikK12K11Θ−,1+ΔK11Θ−,2ζ1ImβipAjpμ−kpμ−1p2+μ−2p2 −4πΘK12K11γ−i1+ΔK11γ−i2ImβipAjp

where Im⋅ is the imaginary value of the variable between the brackets.

All source points whose branch-cut lines cross the domain require the additional line integral to be integrated with intervals $\left(L_{1},L_{2}\right),\;\left(L_{3},L_{4}\right),\ldots ,\;\left(L_{2n-1},L_{2n-2}\right)$. To do so, a strong and robust code is needed to determine all possible intersection points of all source points. Consequently, not only is this inefficient computationally, but it is also extremely difficult to build a strong and robust code to account for all conceivable occurrences of a complex shape. Another significant disadvantage is the requirement to compute internal thermal data along the branch-cut lines involved for evaluation of the extra line integral. However, these internal thermal data can only be obtained after solving the boundary integral equation for thermal analysis, and positions of the internal points are unknown in advance. To do so, the BEM code for computing the associated thermal field must be included into the mechanical analysis code, allowing the computations to be interconnected. This necessitates validating the exact transformation without using the extra line integrals. This procedure is covered in detail in below.

The additional line integral must be integrated over all source points with branch-cut lines that cross the domain.

According to Shiah and Wang [13], we can write

(20) $$Q_{-jjk,tt}^{*}=U_{-ij,k}^{*},$$

where

(21) $$\overset{}{\underset{\Omega }{\int }}\gamma_{-ik}U_{-ij,k}^{*}\Theta d\hat{\Omega }=\overset{}{\underset{s}{\int }}\left(\gamma_{-ik}Q_{-ijk,t}^{*}\Theta -\gamma_{-ik}Q_{-ijk}^{*}\Theta_{-,t}\right)n_{-t}d\hat{S}.\;$$

The derivative of $U_{ij}^{*}$ in Equation (8) yields

(22) U−ij,k*=2ReβimAjmμ−kmz−m.

The generalized variable $z_{-m}$ in the polar coordinates can be expressed as

(23) $$z_{-m}=\hat{r}\left(\mu_{-11}\cos\hat{\theta }+\mu_{-21}\sin\hat{\theta }\right).$$

By substituting Equation (23) into Equation (22), we have

(24) U−ij,k=2rˆReβi1Aj1μ−k1μ−11cosθˆ+μ−21sinθˆ+βi2Aj2μ−k2μ−12cosθˆ+μ−22sinθˆ.

Then, we can re-express $U_{ij,k}^{*}$ as

(25) $$U_{-ij,k}^{*}=\frac{2}{\hat{r}}\overset{b}{\underset{n=-b}{\sum }}D_{-ijk}^{\left(n\right)}e^{\mathrm{in}\hat{\theta }},$$

where b is an integer big enough to ensure series convergence, $\left(\hat{r},\hat{\theta }\right)$ are the polar coordinates, and $D_{-ijk}^{\left(n\right)}$ is the material complex constant. In the current work we considered b=16. 

From Equation (23) and using the theory of Fourier series, we can write $D_{-ijk}^{\left(n\right)}$ as

(26) Dn−jjk=12π∫−ππReβi1Aj1μ−k1μ−11cosθˆ+μ−21sinθˆ+βi2Aj2μ↓2μ−12cosθˆ+μ−22sinθˆe−inθˆdθˆ.

Then, $Q_{-ijk}^{*}$ which satisfies Equation (20) can be expressed as

(27) $$Q_{-ijk}^{*}\left(\hat{r},\hat{\theta }\right)=\hat{r}\rho_{-ijk}\left(\hat{\theta }\right).$$

Substituting Equation (23) into Equation (20), we get

(28) $$\frac{d^{2}\rho_{-ijk}\left(\hat{\theta }\right)}{d{\hat{\theta }}^{2}}+\rho_{-ijk}\left(\hat{\theta }\right)=2\overset{b}{\underset{n=-b}{\sum }}D_{-ijk}^{\left(n\right)}e^{\mathrm{in}\hat{\theta }}.$$

From Equation (28), $\rho_{-ijk}\left(\hat{\theta }\right)$ is determined to be

(29) $$\rho_{-ijk}\left(\hat{\theta }\right)=\overset{b}{\underset{n=-b}{\sum }}\frac{2}{\left(1-n^{2}\right)}D_{-ijk}^{\left(n\right)}e^{\mathrm{in}\hat{\theta }},$$

when $n=\pm 1,\rho_{-ijk}\left(\hat{\theta }\right)$ is not properly defined in Equation (29). As a result, the Fourier series of $U_{-ij,k}^{*}$ is split into two halves—one for n≠ ±1 and one for n=±1.

Thus, we can write $U_{-ij,k}^{*}$ as

(30) $$U_{-ij,-k}=\frac{2}{\hat{r}}\left(D_{-ijk}^{\left(1\right)}e^{i\hat{\theta }}+D_{-ijk}^{\left(-1\right)}e^{-i\hat{\theta }}\right),$$

where $Q_{-ijk}^{*}$ can be expressed as

(31) $$Q_{-ijk}^{*}\left(\hat{r},\hat{\theta }\right)=\hat{r}\ln\hat{r}\lambda_{-ijk}\left(\hat{\theta }\right).$$

Substituting Equation (31) into Equation (20), we obtain

(32) $$\lambda_{-ijk}\left(\hat{\theta }\right)+\ln\hat{r}\left(\frac{d^{2}\lambda_{-ijk}\left(\hat{\theta }\right)}{d{\hat{\theta }}^{2}}+\lambda_{-ijk}\left(\hat{\theta }\right)\right)=2\left(D_{-ijk}^{\left(1\right)}e^{i\hat{\theta }}+D_{-ijk}^{\left(-1\right)}e^{-i\hat{\theta }}\right),$$

where Equation (32) can be satisfied under the following condition:

(33) $$\lambda_{-ijk}\left(\hat{\theta }\right)=D_{-ijk}^{\left(-1\right)}e^{i\hat{\theta }}+D_{-ijk}^{\left(-1\right)}e^{-i\hat{\theta }}.$$

Then, by using Equations (27), (29), (31), and (33), we can write $Q_{-ijk}^{*}$ as

(34) $$\begin{array}{cc} Q_{-ijk}^{*}\left(\hat{r},\hat{\theta }\right)=\hat{r} & \overset{b}{\underset{\underset{n\neq \pm 1}{n=-b}}{\sum }}\frac{2D_{-ijk}^{\left(n\right)}}{\left(1-n^{2}\right)}e^{in\hat{\theta }}+\hat{r}\ln\hat{r}\left(D_{-ijk}^{\left(1\right)}e^{i\hat{\theta }}+D_{-ijk}^{\left(-1\right)}e^{-i\hat{\theta }}\right) \end{array},$$

where $D_{-ijk}^{\left(n\right)}$ is computed using Equation (26).

The explicit expression of $Q_{-ijk,t}^{*}$ may be written as follows [13]:

(35) $$\begin{array}{cc} Q_{-ijk,1}^{*}= & \overset{b}{\underset{\underset{n\neq \pm 1}{n=-b}}{\sum }}\frac{2D_{-jjk}^{\left(n\right)}}{\left(1-n^{2}\right)}\left(\cos\theta -\mathrm{insin}\theta \right)e^{\mathrm{in}\hat{\theta }}\\ & +\ln\hat{r}\left(D_{-ijk}^{\left(1\right)}+D_{-ijk}^{\left(-1\right)}\right)+\cos\hat{\theta }\left(D_{-ijk}^{\left(1\right)}e^{i\hat{\theta }}+D_{-ijk}^{\left(-1\right)}e^{-i\hat{\theta }}\right), \end{array}\;$$

(36) $$\begin{array}{cc} Q_{-ijk,2}^{*}= & \overset{b}{\underset{\underset{n\neq \pm 1}{n=-b}}{\sum }}\frac{2D_{-ijk}^{\left(n\right)}}{\left(1-n^{2}\right)}\left(\sin\hat{\theta }+\mathrm{in}\;\cos\hat{\theta }\right)e^{\mathrm{in}\hat{\theta }}\\ & +\ln\hat{r}\left(D_{-ijk}^{\left(1\right)}-D_{-ijk}^{\left(-1\right)}\right)+\sin\theta \left(D_{-ijk}^{\left(1\right)}e^{i\hat{\theta }}+D_{-ijk}^{\left(-1\right)}e^{-i\hat{\theta }}\right). \end{array}$$

## 4. Numerical Results and Discussion

To validate the analysis, we consider a thick hollow disc with four inside holes [13]. The current problem was analyzed using FlexPDE 7 which is based on the finite element method (FEM). For the elastic boundary conditions, the exterior surface was fully constrained, although the interior was traction-free in all directions. Moreover, insulation was required on all surfaces of the four inside holes.

The thermoelastic constants of monoclinic graphite–epoxy can be written as follows:

Elasticity tensor

(37) $$C_{pjkl}=\left[\begin{array}{cc} \begin{array}{ccc} 430.1 & 130.4 & 18.2\\ 130.4 & 116.7 & 21.0\\ 18.2 & 21.0 & 73.6 \end{array} & \begin{array}{ccc} 0\; & 0 & \;201.3\\ 0\; & 0 & \;70.1\\ 0\; & 0 & \;2.4 \end{array}\\ \begin{array}{ccc} 0 & 0 & 0\\ 0 & 0 & 0\\ 201.3 & 70.1 & \;2.4 \end{array} & \begin{array}{ccc} 19.8 & -8.0\; & \;0\;\\ -8.0 & 29.1 & 0\;\\ \;0 & 0\; & 147.3\; \end{array} \end{array}\right]\mathrm{GPa};$$

Mechanical temperature coefficient

(38) $$\beta_{pj}=\left[\begin{array}{ccc} 1.01 & 2.00 & 0\\ 2.00 & 1.48 & 0\\ 0 & 0 & 7.52 \end{array}\right]\cdot 10^{6}\;N/{\mathrm{km}}^{2};$$

Thermal conductivity tensor

(39) $$k_{pj}=\left[\begin{array}{ccc} 5.2 & 0 & 0\\ 0 & 7.6 & 0\\ 0 & 0 & 38.3 \end{array}\right]\;W/\mathrm{km};$$

Mass density $\rho =7820\;\mathrm{kg}/m^{3}$;

Heat capacity $c=461\;J/\left(\mathrm{kg}\;K\right)$.

**Figure 1:**
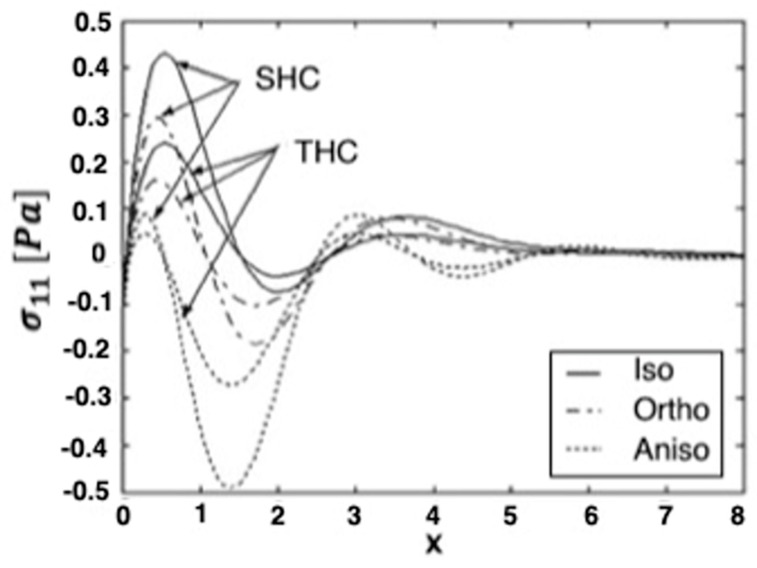
Variation of the thermal stress σ_11_ along *x*-axis in the transient and steady-state heat con-duction for isotropic, orthotropic, and anisotropic cases.

**Figure 2:**
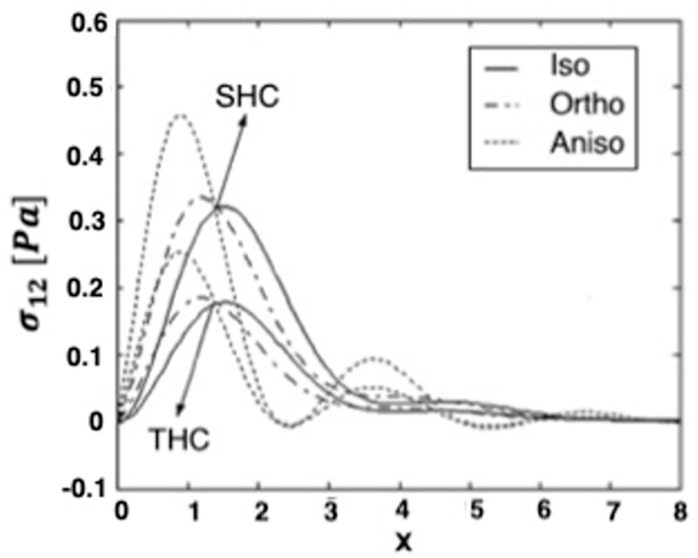
Variation of the thermal stress σ_12_ along *x*-axis in the transient and steady-state heat con-duction for isotropic, orthotropic, and anisotropic cases.

**Figure 3:**
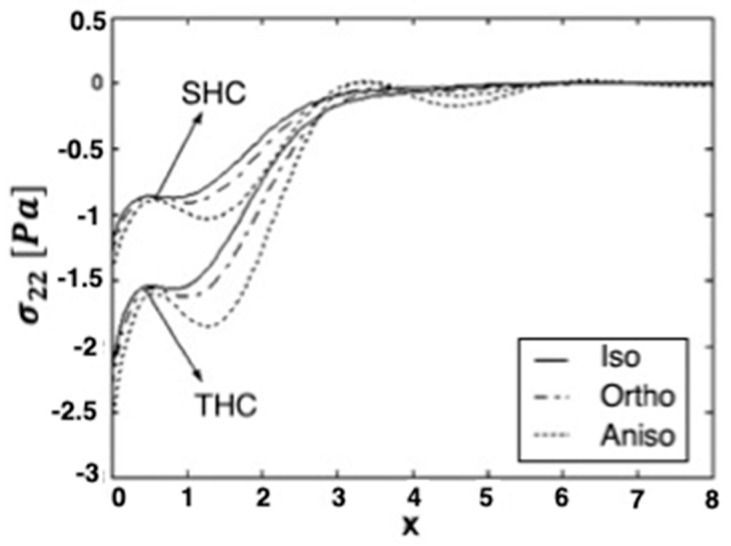
Variation of the thermal stress σ_22_ along *x*-axis in the transient and steady-state heat con-duction for isotropic, orthotropic, and anisotropic cases.

Figure 1, Figure 2 and Figure 3 show the effect of transient and steady-state heat conduction on the quasi-static thermal stresses $\sigma_{11}$, $\sigma_{12}$, and $\sigma_{22}$ along the x-axis in the transient, as well as the steady-state heat conduction for isotropic, orthotropic, and anisotropic cases.

Figure 1 depicts the distribution of the quasi-static thermal stress component $\sigma_{11}$ in the context of the isotropic, orthotropic, and anisotropic materials. It demonstrates that, in the context of the three considered materials, $\sigma_{11}$ increases at first to a maximum value, before decreasing to a minimum value in the transient heat conduction (THC) and steady-state heat conduction (SHC). It shows that, in the context of the three considered materials, $\sigma_{11}$ converges to zero with increasing distance *x*.

Figure 2 depicts the distribution of the quasi-static thermal stress component $\sigma_{12}$, demonstrating that the stress component $\sigma_{12}$ reaches a zero value. It shows that, in the context of isotropic and orthotropic materials, $\sigma_{12}$ increases to a maximum value, before sharply decreasing in the transient and steady-state heat conduction. However, in the context of the anisotropic material,$\;\sigma_{12}$ increases to a maximum value, before decreasing to a minimum value in the transient and steady-state heat conduction. In the context of the three materials, $\sigma_{12}$ converges to zero with increasing distance x**,** while the values of the stress component $\sigma_{12}$ in the transient heat conduction are higher than those in the steady-state heat conduction.

Figure 3 shows the distribution of the quasi-static thermal stress component $\sigma_{22}$ in the context of the three materials; it begins with a negative value decrease in the transient and steady-state heat conduction. The values of $\sigma_{22}$ in the context of the isotropic and orthotropic materials increase initially in the range 0≤x≤0.7, before decreasing in the range 0.7≤x≤1, and again increasing to a maximum value in the transient and steady-state heat conduction. However, in the context of the anisotropic material, $\sigma_{22}$ increases at first, before decreasing to a minimum value in the transient and steady-state heat conduction The values of the stress component $\sigma_{22}$ in the transient heat conduction are higher than those in the steady-state heat conduction and converge to zero with increasing distance x in the transient and steady-state heat conduction.

**Figure 4:**
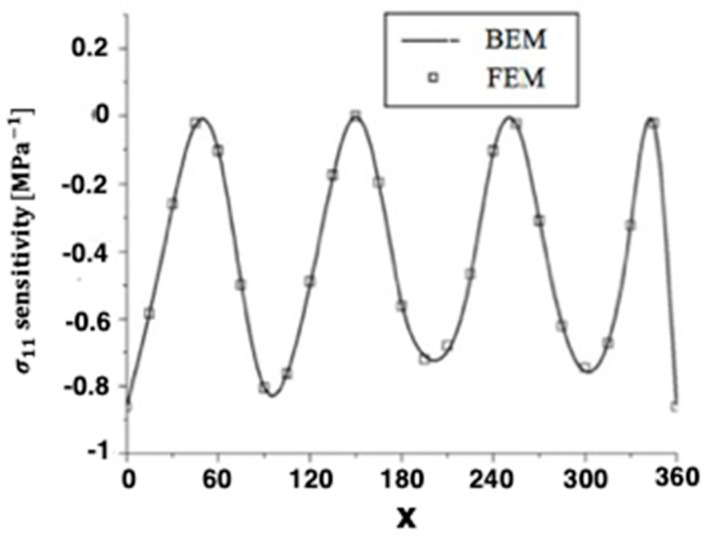
Variation of the thermal stress σ_11_ sensitivity along *x*-axis of the disc inner surface for BEM and FEM.

**Figure 5:**
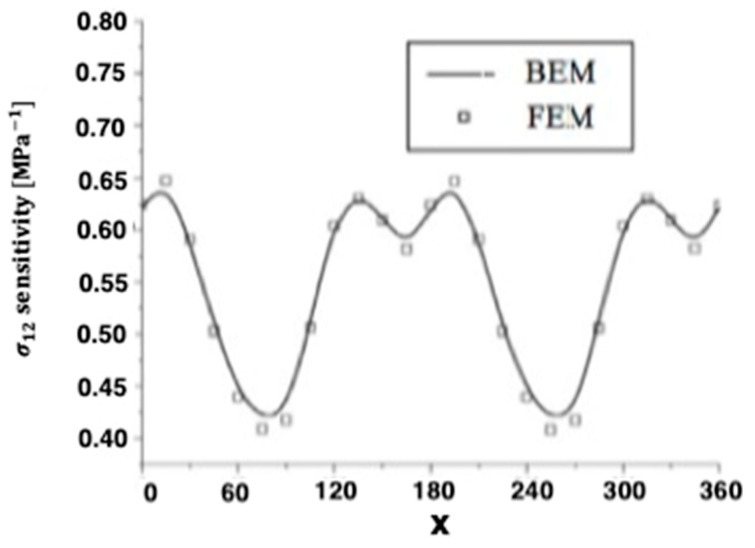
Variation of the thermal stress σ_12_ sensitivity along *x*-axis of the disc inner surface for BEM and FEM.

Quasi-static thermal stresses $\sigma_{11}$ and $\sigma_{12}$ of anisotropic thermoelastic metal and alloy discs with holes were calculated on the inner surface of the disc, as well as the other four holes inside. Figure 4 and Figure 5 show the sensitivity variations of the thermal stresses $\sigma_{11}$ and $\sigma_{12}$ along the x-axis in the transient and steady-state heat conduction for isotropic, orthotropic, and anisotropic cases.

**Figure 6:**
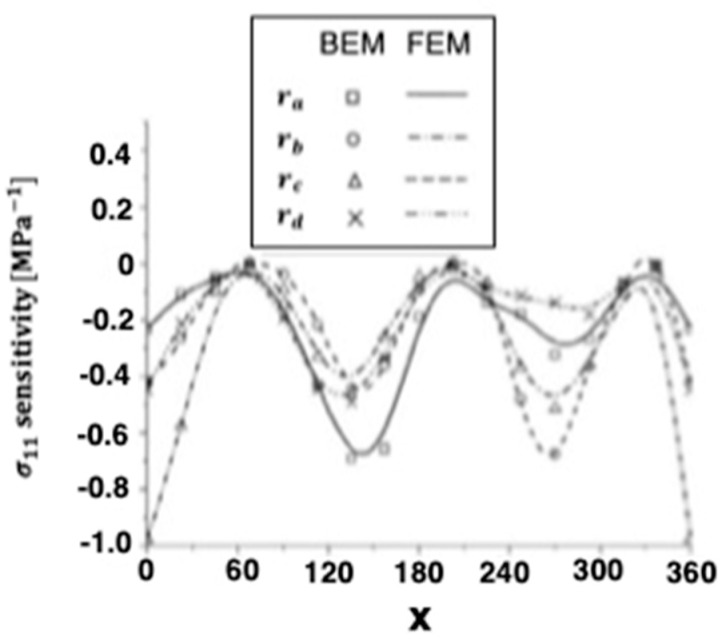
Variation of the thermal stress σ_11_ sensitivity along *x*-axis on the holes inside for BEM and FEM.

**Figure 7:**
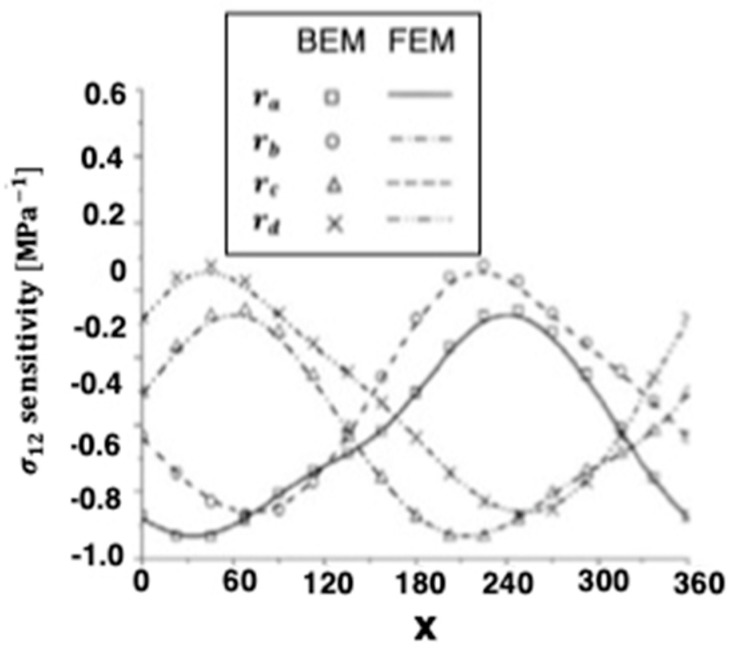
Variation of the thermal stress σ_12_ sensitivity along *x*-axis on the holes inside for BEM and FEM.

The numerical results of the quasi-static thermal stresses $\sigma_{11}$ and $\sigma_{12}$ for the four holes are presented in Figure 6 and Figure 7 for BEM and FEM analysis of anisotropic thermoelastic metal and alloy discs with holes. It can be seen from these figures that the BEM results are in very good agreement with the FEM results.

**Figure 8:**
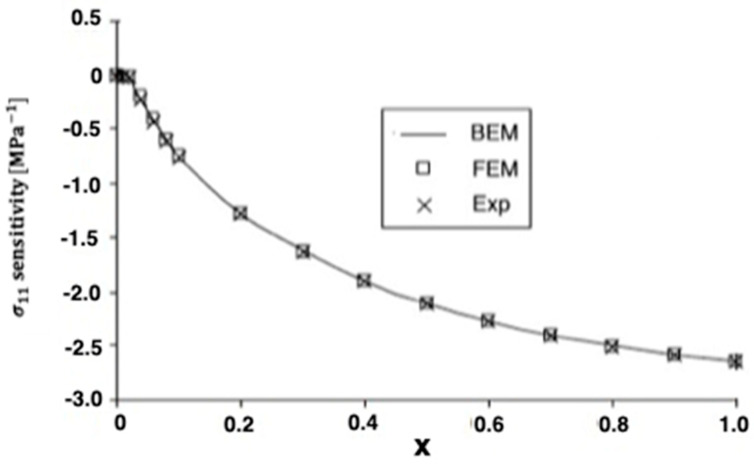
Variation of the thermal stress σ_11_ sensitivity along *x*-axis on the holes inside for BEM, FEM, and Exp.

**Figure 9:**
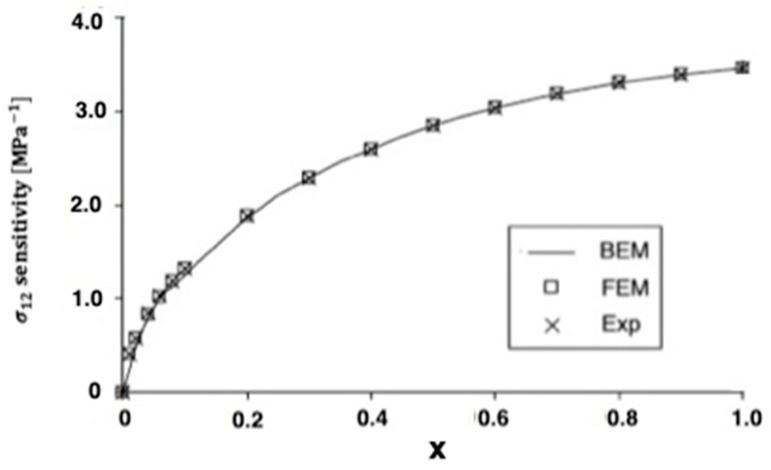
Variation of the thermal stress σ_12_ sensitivity along *x*-axis on the holes inside for BEM, FEM, and Exp.

The advantages of spark plasma sintering (SPS) over conventional hot pressing (HP) or hot isostatic pressing (HIP), such as reduced sintering time and temperatures, minimize grain growth and frequently result in improved mechanical, physical, or optical properties. Therefore, SPS has been used successfully to manufacture a variety of metals. The thermoelastic problem studied in Wang et al. [14] can be treated as a special case of our study of analysis of anisotropic thermoelastic metal and alloy discs with holes. To analyze the thermal stress sensitivity distribution in the SPS process, we used the same model as in [14] with the boundary element method (BEM). In this special case under consideration, the numerical results for the quasi-static thermal stresses $\sigma_{11}$ and $\sigma_{12}$ are shown in Figure 8 and Figure 9. It can be seen from these figures that our BEM results are in excellent agreement with the FEM results and experimental (Exp) results of Wang et al. [14]. We refer the interested readers to [15,16,17,18,19,20,21,22] for further references.

Table 1 shows a comparison of the computer resources needed in the analysis of anisotropic thermoelastic metal and alloy discs, for BEM with additional line integrals (Case 1) versus BEM without additional line integrals (Case 2). It can be seen from this table that the proposed BEM without additional line integrals is more accurate and efficient than the BEM with additional line integrals.

## 5. Conclusions

The examination of the numerical results and figures enables us to make some concluding remarks:

1. The current research has received a lot of attention because of its practical applications in fields such as astronautics, geomechanics, earthquake engineering, nuclear reactors, material science, and other industrial applications.
2. Because the proposed boundary element approach only needs to solve the boundary unknowns, it solves problems faster and more accurately than domain approaches while also minimizing the solver’s processing costs.
3. Avoiding the use of additional line integrals by using branch-cut redefinitions in the current study plays a significant role in all the physical quantities and their design sensitivities.
4. The current results were validated against the numerical and experimental results obtained through other methods previously validated. It should be noted that the BEM results are in excellent agreement with the FEM and experimental results, confirming the accuracy of the BEM technique.
5. Current numerical results for our complex and general problem may be of interest to engineers and material science researchers, as well as those working on the development of anisotropic thermoelastic metal and alloy discs with holes.
6. It can be concluded from analysis results that the proposed technique is more efficient than other techniques in the literature for analyzing anisotropic thermoelastic metal and alloy discs with holes.
7. The numerical results show that the proposed BEM is ideal for analyzing anisotropic thermoelastic metal and alloy discs with holes.

## Figures and Tables

**Table 1:** Comparison of computer resources required for BEM with additional line integrals (Case 1) and BEM without additional line integrals (Case 2).

	FEM	BEM (Case 1)	BEM (Case 2)
CPU time (min)	28	24	4
Memory (MB)	26	22	1
Disc space (MB)	38	32	0
Accuracy of results (%)	2.2	2.1	1.1

## Data Availability

All data generated or analysed during this study are included in this published article.
